# The Expression of the SLIT–ROBO Family in Adult Patients with Acute Myeloid Leukemia

**DOI:** 10.1007/s00005-019-00535-8

**Published:** 2019-02-28

**Authors:** Aleksandra Gołos, Dorota Jesionek-Kupnicka, Lidia Gil, Marcin Braun, Mieczyslaw Komarnicki, Tadeusz Robak, Agnieszka Wierzbowska

**Affiliations:** 1 Department of Hematology, Medical University, Lodz, Poland; 2 Department of Hematology, Institute of Hematology and Transfusion Medicine, Warsaw, Poland; 3 Department of Pathology, Medical University, Lodz, Poland; 4 Department of Hematology, University of Medical Sciences, Poznan, Poland; 5 Postgraduate School of Medicine, Medical University of Warsaw, Warsaw, Poland

**Keywords:** SLIT protein, ROBO protein, Angiogenesis, Acute myeloid leukemia, Nerve tissue proteins

## Abstract

**Introduction:**

SLIT–ROBO is a ligand–receptor family of neuronal guidance cues that has been involved in pathological and physiological angiogenesis. SLIT–ROBO expression is altered in many tumours. However, no data exist about the role of the whole family in acute myelogenous myeloid leukemia (AML).

**Purpose:**

Herein, we assessed the expression of all SLIT–ROBO family in bone marrow (BM) biopsy of AML patients and control group on both protein and RNA levels.

**Methods:**

The paraffin-embedded tissue blocks were subjected to immunohistochemistry for SLIT1, SLIT2, SLIT3, ROBO1, ROBO2, ROBO3, and ROBO4. Microvessel density (MVD) was evaluated by CD34 immunohistochemistry. An in silico analysis using The Cancer Genome Atlas data repository was conducted for assessment of RNA level.

**Results:**

Acute myeloid leukemia patients were generally high expressers of ROBO1 and ROBO2 compared to the controls (*p* < 0.0001, *p* < 0.001, respectively). In contrast, low expression of SLIT1, SLIT2, and SLIT3 ligands has been noted more commonly in AML than in control BM samples (*p* < 0.0001, *p* = 0.003, and *p* = 0.001, respectively). ROBO4 expression correlated with MVD. The in silico analysis showed a poor prognostic value of high ROBO3 and low SLIT2 RNA levels (*p* = 0.0003 and *p* = 0.0008, respectively), as well as high ROBO3 and ROBO4 RNA levels in cytogenetic poor risk groups of patients (*p* = 0.0029 and *p* = 0.0003, respectively).

**Conclusions:**

These data indicate that SLIT–ROBO family members play a role in the biology of AML. Low expression of SLIT in BM of AML patients may suggest its expression alterations in AML. Increased expression of ROBO1 and ROBO2 in AML patients suggests their participation in AML pathogenesis.

## Introduction

SLIT and their ROBO receptors are members of the axon guidance molecule family that have been identified to play a crucial role in the development of nervous system of vertebrates and invertebrates (Kidd et al. 1998). The family consists of four receptors ROBO (ROBO1, ROBO2, ROBO3, and ROBO4) and three ligands SLIT (SLIT1, SLIT2, SLIT3). The SLITs were first described as a ligand for ROBO in *Drosophila melanogaster* (Seeger et al. 1993). ROBOs belong to the immunoglobulin-like superfamily of transmembrane receptors (Hohenester 2008). There is evidence that this signaling pathway plays a role in organogenesis, cell migration and apoptosis (Grieshammer et al. 2004; Dickinson et al. 2010, 2008) It has been also found that the guidance cues are involved in both, physiological and pathological angiogenesis (Carmeliet and Tessier-Lavigne 2005; Brose and Tessier-Lavigne 2000). Moreover, many studies suggest its involvement in tumorigenesis (Wang et al. 2003; Dallol et al. 2002).

SLIT–ROBO expression has been described in many types of human cancers, yet its role in the biology of the disease remains controversial. It has been postulated that SLIT–ROBO may function as a tumor suppressor system, i.e. in cervical, breast, non-small cell lung and ovarian cancers (Singh et al. 2007; Sharma et al. 2007; Görn et al. 2005; Dai et al. 2011). Conversely, increased expression of SLIT–ROBO family members has been reported in prostate, colorectal, hepatocellular, and endometrial carcinomas (Latil et al. 2003; Gröne et al. 2006; Ito 2006; Ma et al. 2010) Little is known about the expression of SLIT–ROBO proteins in hematological malignancies. However, ROBO4 is known to be expressed on the surface of hematopoietic stem cells (HSC), and takes part in niche reaching by HSC (Smith-Berdan et al. 2011). To date, there has been little published data concerning the role of the SLIT–ROBO pathway in the biology of AML.

The aim of the present study was to assess the expression of all the proteins from SLIT–ROBO family (SLIT1, SLIT2, SLIT3, ROBO1, ROBO2, ROBO3, and ROBO4) in the BM biopsy of AML patients by immunohistochemical staining. The relationship between SLIT–ROBO protein expression and bone marrow angiogenesis was also investigated. Finally, we conducted a comprehensive analysis using The Cancer Genome Atlas data repository to assess the expression of ROBO–SLIT also on the RNA level. To our best knowledge, this is the first study to investigate the whole family on both protein and RNA levels in acute myeloid leukemia.

## Materials and Methods

### Ethic Statements

All the blood and BM samples were collected from patients after obtaining their written informed consent. The study was approved by the Ethics Committee of the Medical University of Lodz, Poland (RNN/2/13/KE).

### Patients

Seventy-nine newly diagnosed AML patients, median age 59 years (range 18–87 years), entered the study between 2006 and 2013. Acute promyelocytic leukemia patients were excluded. The patients were treated in the Hematology Department of the Medical University of Lodz (52 patients), and in the Department of Hematology of the University of Medical Sciences, Poznan (27 patients). The diagnosis was based on standard morphological, cytochemical, immunophenotypic, and cytogenetic criteria (Dohner et al. 2010). The cytogenetic risk stratification was made according to the SWOG (South Western Oncology Group) criteria (Slovak et al. 2000). The ECOG scale was used to define the general assessment (Oken et al. 1982).

All of the patients eligible for intensive chemotherapy were treated according to the 3 + 7 or DAC protocols (Holowiecki et al. 2004). Patients aged over 60 years, with comorbidities and poorer performance status (ECOG ≥ 2), were given hypomethylating agents such as azacitidine or decitabine, or low-dose cytarabine. Patients not eligible for any chemotherapy were given palliative care with hydroxyurea and/or best supportive care (BSC). The control group was composed of 23 patients with newly diagnosed lymphoma without BM involvement. The median age of the group was 52 years (range 21–76 years). The clinical characteristics of both groups are presented in Table 1.

**Table 1:** Clinical characteristics of the patients and the control group

Characteristics	Value
*AML group*	
Number (*n*)	79
Age (year)	
Me	59
Range	18–87
Sex (female/male)	37/42
WBC (× 10^9^/L)	
Me	4.48
Range	0.54–252
Hb (g/dL)	
Me	8.4
Range	3.0–14.3
PLT (x10^9^/L)	
Me	54
Range	6–586
LDH (U/L)	
Me	299
Range	163–3350
BM blasts	(%)
Me	47
Range	20–100

Cytogenetic risk	*n* (%)
Favorable	6 (7.6%)
Intermediate	37 (46.8%)
Unfavorable	25 (31.6%)
Unknown	11 (13.9%)

Treatment	*n* (%)
Intensive	38 (48.1%)
Non-intensive	30 (38%)
HMA	17 (21.5%)
LDAC	13 (16.5%)
BSC	11 (13.9%)

Number (*n*)	23
*Control group*	
Age (year)	
Me	52
Range	21–76
Sex (female/male)	10/13

Diagnosis	*n* (%)
DLBCL	10 (43%)
HL	4 (17%)
MCL	3 (13%)
Others	6 (26%)

*Me* median, *WBC* white blood cells, *Hb* hemoglobin level, *PLT* platelets, *LDH* lactate dehydrogenase, *SWOG* Southwestern Oncology Group, *HMA* hypomethylating agents, *LDAC* low-dose arabinoside cytosine, *DLBCL* diffuse large B-cell lymphoma, *HL* Hodgkin lymphoma, *MCL* mantle cell lymphoma

### Immunohistochemistry

Immunohistochemical staining was performed on paraffin-embedded 4-µm sections. The sections were dewaxed in 98% xylene solution (three times for 3 min) before being dehydrated in 96% alcohol (three times for 1 min) and rinsed in water. Antigen retrieval was performed by placing the slides in a bath of target retrieval solution (pH 6), (K8805, DAKO, Denmark), and boiling for 15 min in a 360-W microwave oven. The volume of fluid was topped up, and the slides were left to cool to 60 °C at room temperature. After rinsing the specimens in distilled water, the endogenous peroxidase was quenched in a peroxidase-blocking solution (S202386-2, DAKO, Denmark) for 10 min. The slides were washed in Tris-buffer (S3006, DAKO, Denmark) for 5 min at room temperature. The sections were incubated for 24 h in 4 °C with the primary monoclonal antibodies to the following: SLIT1 (sc-28944, Santa Cruz Biotechnology, USA, 1:10, pH 6), SLIT2 (sc-16619, Santa Cruz Biotechnology, USA, 1:50, pH 6), SLIT3 (sc-31597, Santa Cruz Biotechnology, USA, 1:100, pH 6), ROBO1 (sc-16611, Santa Cruz Biotechnology, USA, 1:100, pH 6), ROBO2 (sc-16615, Santa Cruz Biotechnology, USA, 1:100, pH 6), ROBO3 (bs-11476R, Bioss, USA, 1:500, pH 6), and ROBO4 (sc-166872, Santa Cruz Biotechnology, USA, 1:10, pH 6). Antibody diluent (S080983-2, DAKO, Denmark) was used for all solutions. After washing in Tris-buffer, the sections were incubated for 30 min with a biotinylated secondary antibody (K069011-2, DAKO, Denmark) and rinsed in Tris-buffer again. The slides were subsequently incubated with horseradish-conjugated streptavidin for 30 min (K069011-2, DAKO, Denmark) and washed in Tris-buffer for 5 min. They were then treated for 5 min in 3,3-diaminobenzidine (K346811-2, DAKO, Denmark), rinsed in distilled water, counterstained in hematoxylin for 3 min (BIO-05-M06002, Biooptica, Italy), and washed in tap water. The slides were then dehydrated in 96% alcohol three times for 1 min and in 98% xylene three times for 1 min. Coverlips were mounted with DPX (Histokit). The validity of immunohistochemical results requires similar conditions of reactions for all tests and appropriate positive and negative controls. As a positive control, we used spleen and tonsil sections, without neoplastic lesions. As negative controls, we used isotype-specific immunoglobulins as a substitute for the primary antibody (Hewitt et al. 2014). The staining intensity of each of the protein was semi-quantitatively analyzed. The percentage of stained cells (0–4 pts) and staining intensity (1–3 pts) was scored, and the points were added to give a final result according to IHC standards (Perrone et al. 2006) This encoding system is presented in Table 2.

**Table 2:** Immunohistochemical scoring system (Perrone et al. 2006)

Quantitative evaluation		Staining intensity		Final evaluation	
% Positive cells	Score	Intensity	Score	Total score	Expression
< 1	0	Weak	1	0–2	Low
1–20	1	Moderate	2	3–5	Intermediate
20–50	2	Strong	3	6–7	High
50–80	3				
> 80	4				

### Evaluation of Angiogenesis

**Fig. 1:**
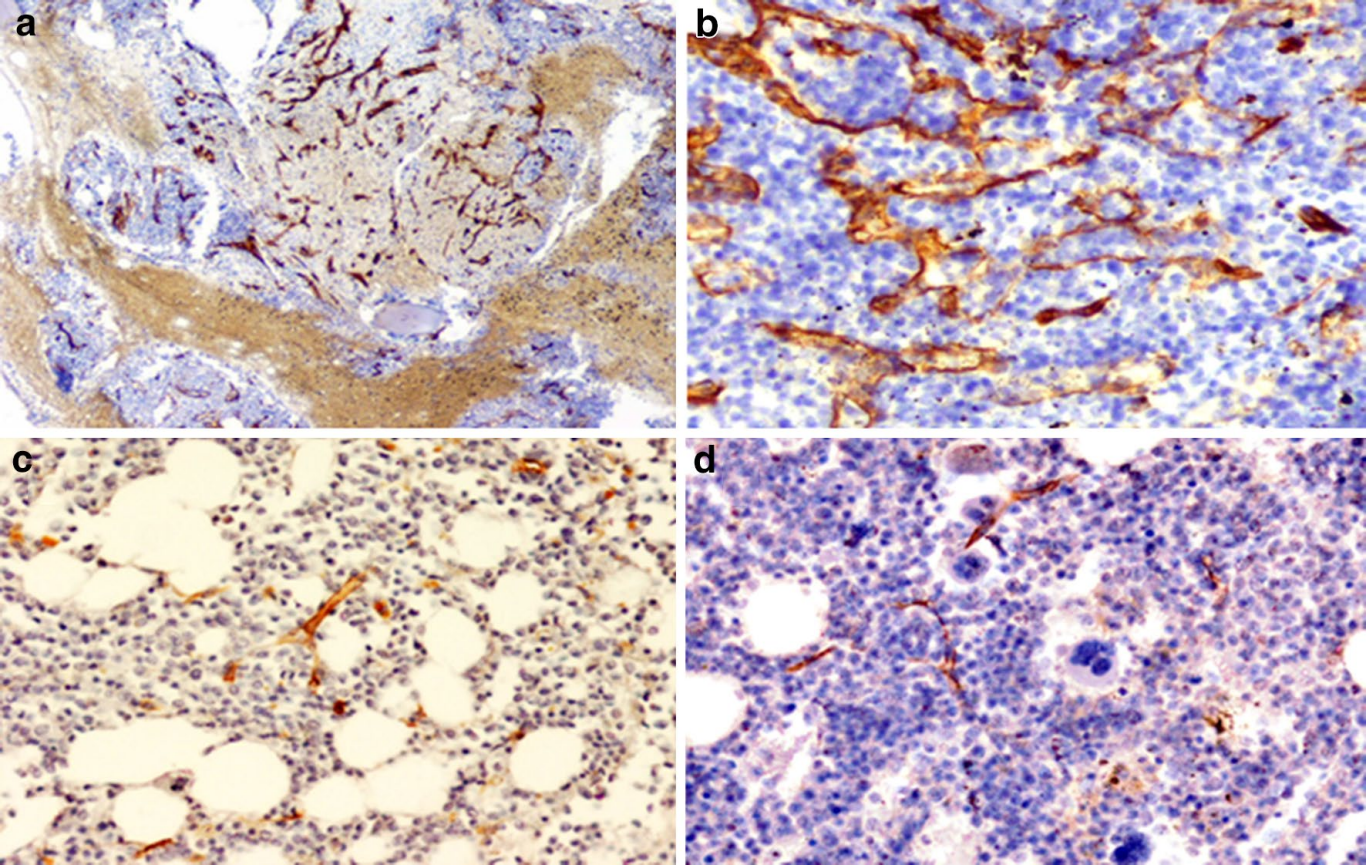
The estimation of microvessel density (MVD) using immunostaining of anti-CD34 antibody: **a** the areas having the highest number of capillaries and small venules (“hot spots”) were selected in low-power field (× 40) **b** then the microvessels were counted in these fields with a magnification of × 200: AML case (**a, b**) and control bone marrow trephine biopsy slides (**c, d**). The CD34-positive blast was considered as cells with nucleoli, on contrary to microvessels with lumen (hematoxylin staining). Magnification × 200

The intensity of angiogenesis was measured quantitatively using MVD calculation. MVD was assessed on CD34-stained slides (M7165, DAKOCytomation, Denmark, 1:50, pH 9) by light microscopy in the areas having the highest number of capillaries with the lumen < 10 µm and small venules without lumen or with the lumen of < 10 µm (“hot spots”). Subsequently, only the microvessels were counted in three chosen fields in each sample (number of vessels/mm^2^) with a magnification of × 200. The MVD was calculated as the mean vessel count from these three fields, as described previously (Vermeulen et al. 1996). The CD34-positive blasts were considered as cells with nucleoli, on contrary to microvessels with lumen (hematoxylin staining) (Fig. 1).

### In Silico Analyses

Apart from the protein level, the RNA expression levels of the selected genes were compared between leukemic cells and normal hematopoietic cells using public repositories. We used BloodSpot database (Bagger et al. 2016), The Cancer Genome Atlas Research Network (TCGA) website with utilization of cBioPortal for Cancer Genomics (Weinstein et al. 2013; Cerami et al. 2012; Ley et al. 2013). Additionally, the analyses for associations between clinicopathological features of AML and RNA expression levels of the selected genes were done using the downloaded data. The expression data were presented as Fragments Per Kilobase of transcript per Million mapped reads upper quartile (FPKM-UQ) as the normalization method.

### Statistical Analysis

The statistical analysis was performed with STATISTICA 10.0 (StatSoft, Inc, OK, USA). The data were expressed as median values and ranges for continuous variables. The medians were compared using the Mann–Whitney *U* test. The chi-squared test was used to investigate the dependence between two categorical variables. The log-rank test was used to assess the impact of variables on overall survival (OS). Survival was plotted using Kaplan–Meier plots. Comparisons and correlations were considered significant if *p* < 0.05.

## Results

### Expression of ROBO Receptors and SLIT Ligands in AML and in the Control Group

Immunohistochemical staining reactions for each of the protein family were analyzed in 79 patients in AML group and 23 in the control group. The patients were divided into low- and high-expresser groups.

**Fig. 2:**
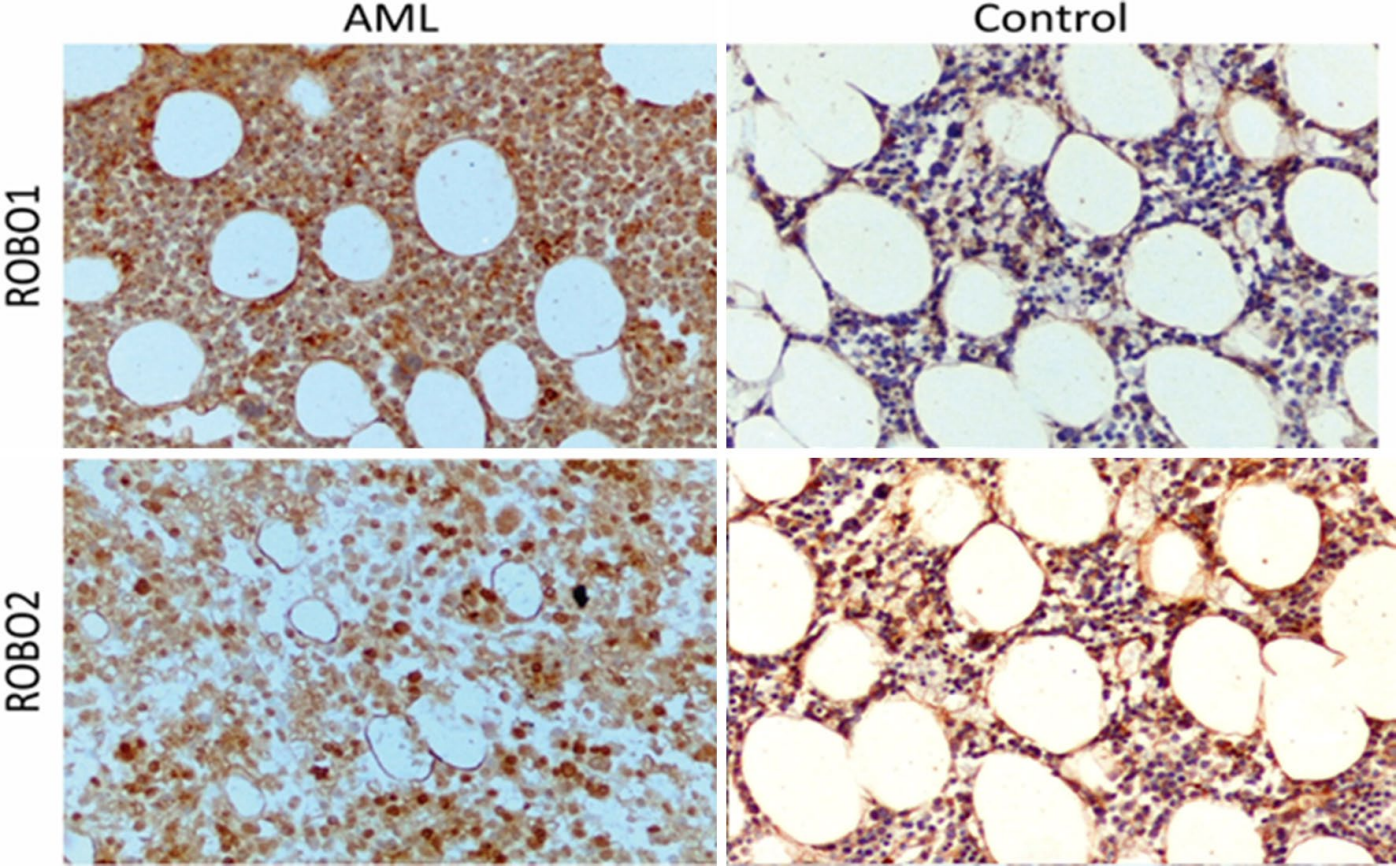
Anti-ROBO1 and -ROBO2 staining for AML and the control group. Magnification × 200

**Fig. 3:**
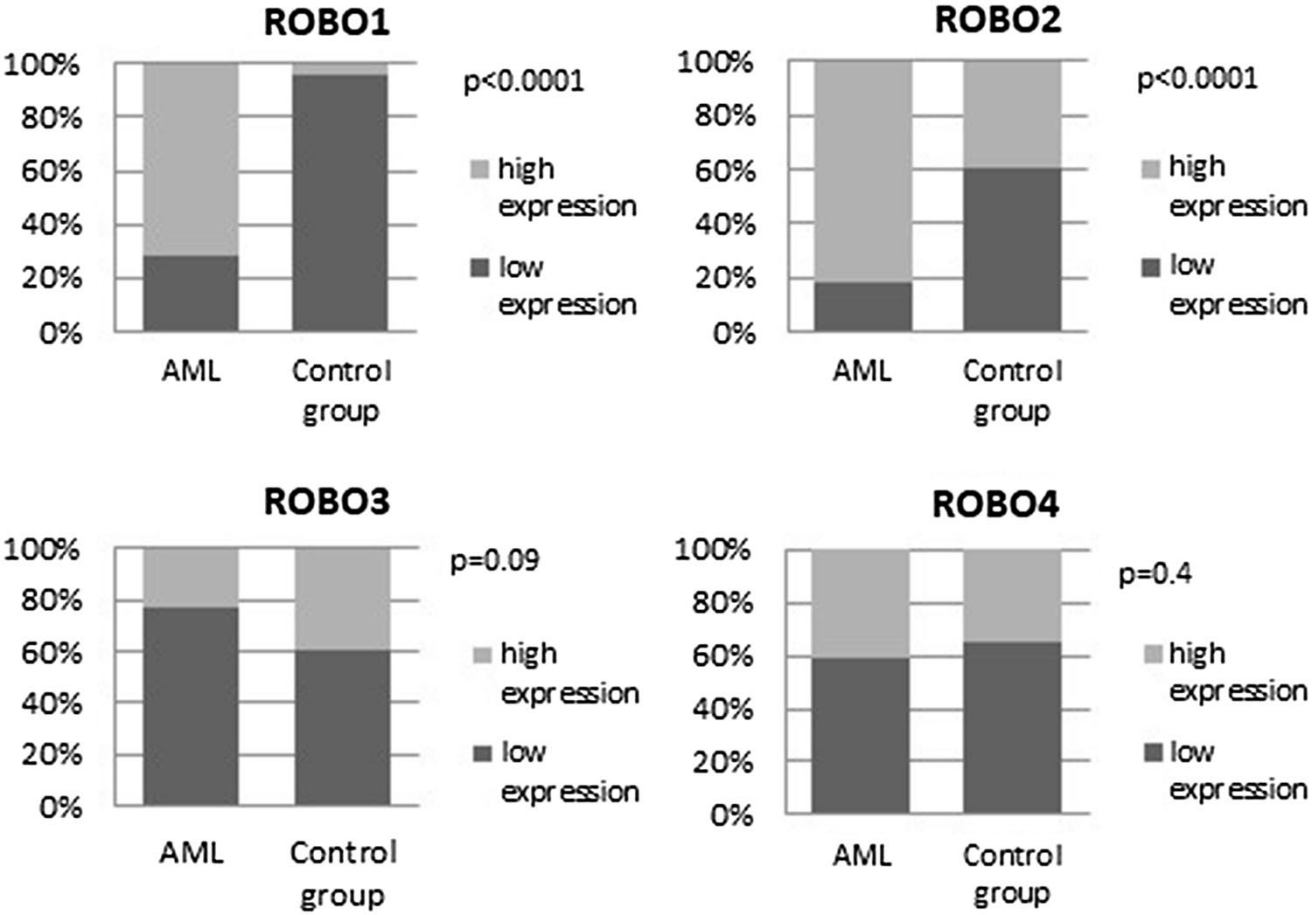
Frequency of low and high expression of ROBO1, ROBO2, ROBO3, and ROBO4 in AML and the control group

It was found that AML patients were generally high expressers of ROBO1, high expression being noted in 72.15% of the AML group compared to 4.35% of controls (*p* < 0.0001), and of ROBO2, with 82.28% of the AML group expressing high ROBO2 compared to 39.13% of controls (*p* < 0.001). The staining reaction for ROBO1 and ROBO2 is presented in Fig. 2. There was a trend toward lower expression of ROBO3 in the AML group than in the controls (77.22% vs 60.87%, respectively, *p* = 0.09). Low expression of ROBO4 was found more frequently in both, AML and the control group (59.49% vs 65.22%). However, the difference did not reach the statistical significance (*p* = 0.4). The frequencies of ROBO expression in both groups are presented in Fig. 3.

**Fig. 4:**
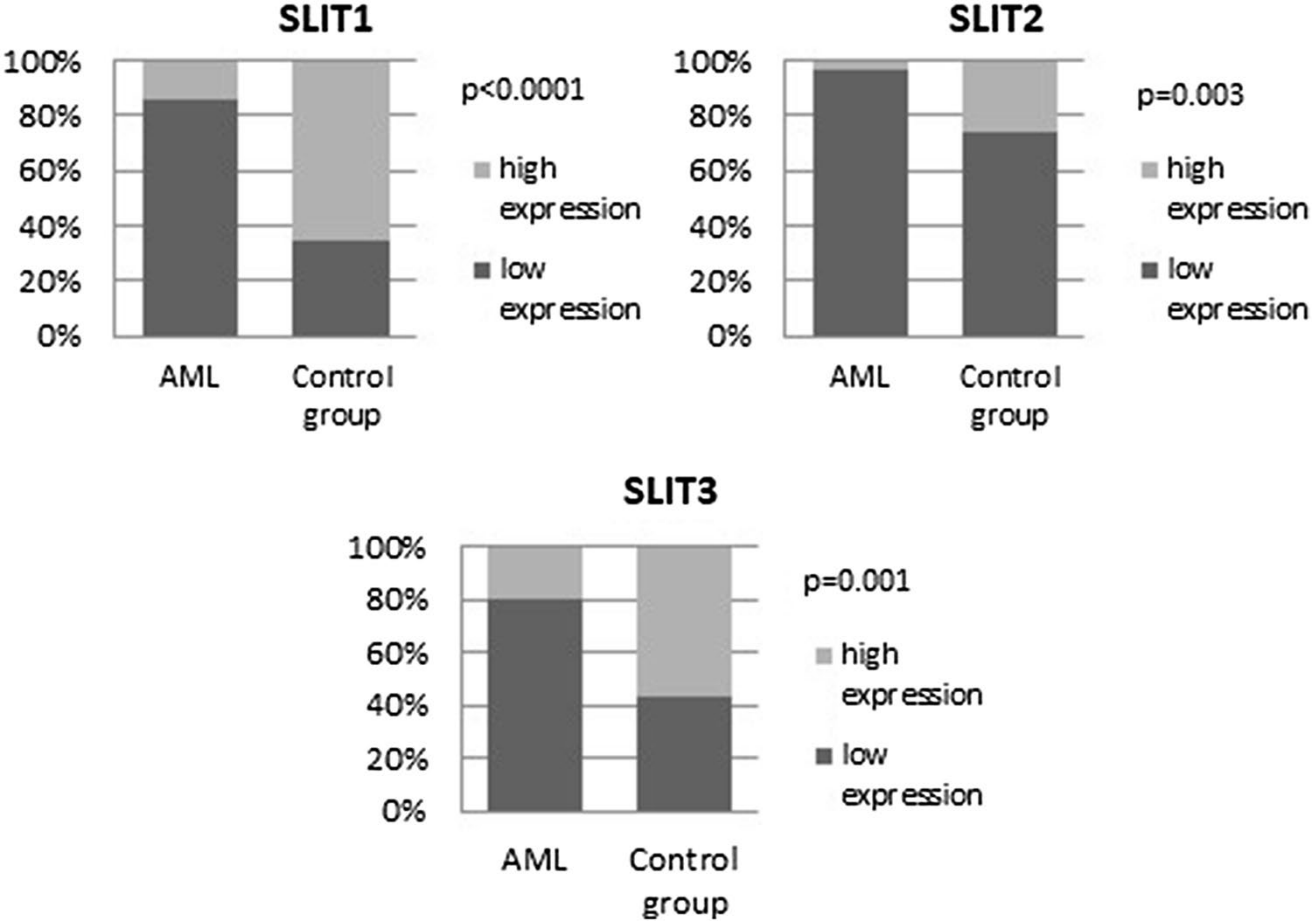
Frequency of low and high SLIT1, SLIT2, and SLIT3 expression in AML and the control group

**Fig. 5:**
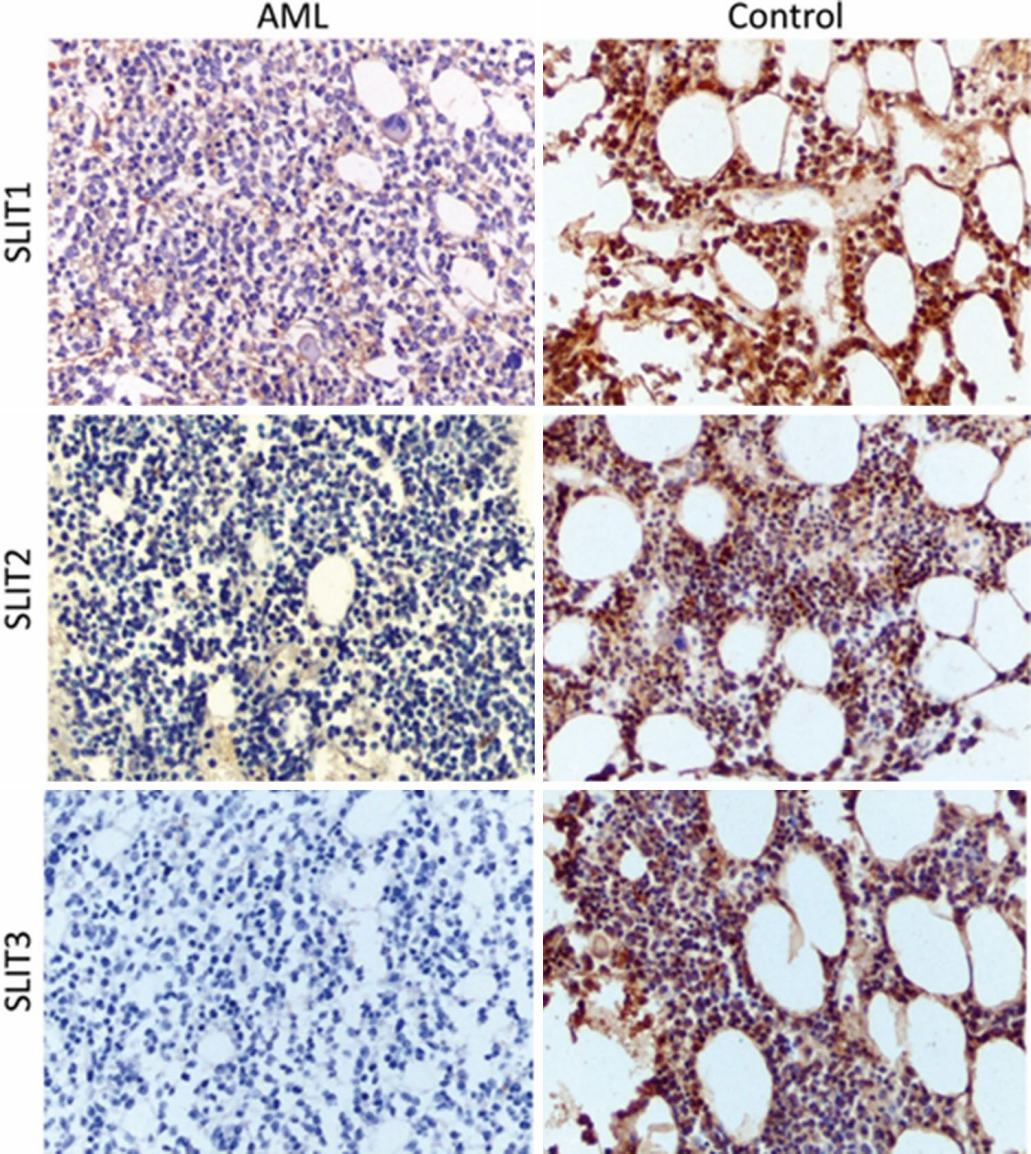
Anti-SLIT1, -SLIT2, and -SLIT3 staining in AML and the control group

Low SLIT expression was observed more commonly in the AML group than controls. Low expression of SLIT1 was observed in 86.08% of AML group but in 34.78% in the control group (*p* < 0.0001), and low SLIT2 expression was observed in 96.2% of AML patients and in 73.91% of controls (*p* = 0.003). Finally, low SLIT3 expression was seen in 79.75% of AML patients and in 43.48% of the control group (*p* = 0.001), (Fig. 4). The staining reactions for SLITs are presented in Fig. 5.

**Fig. 6:**
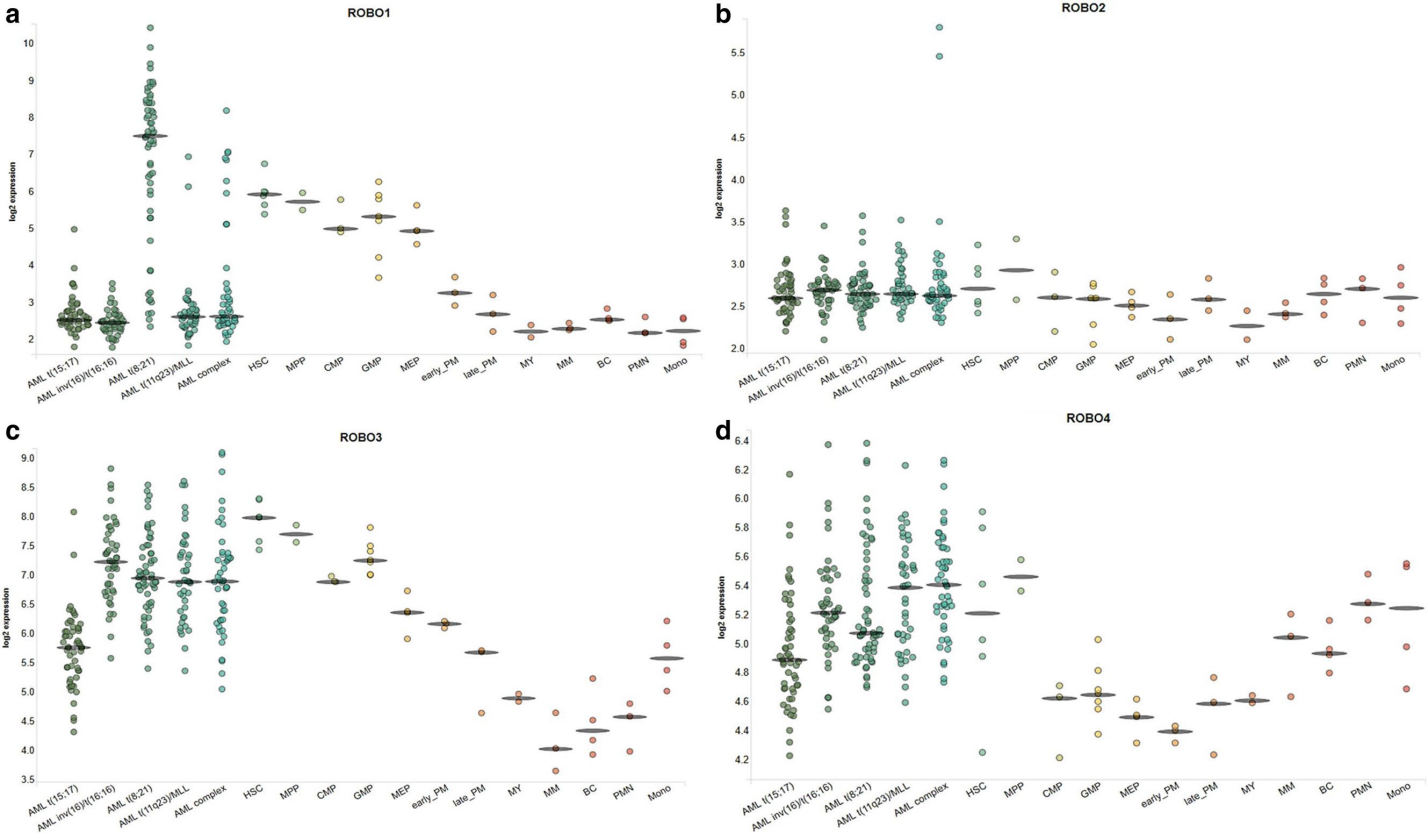
The comparison of mRNA levels between leukemic and normal hematopoietic cells using BloodSpot tool; **a** ROBO1, **b** ROBO2, **c** ROBO3, **d** ROBO4

**Fig. 7:**
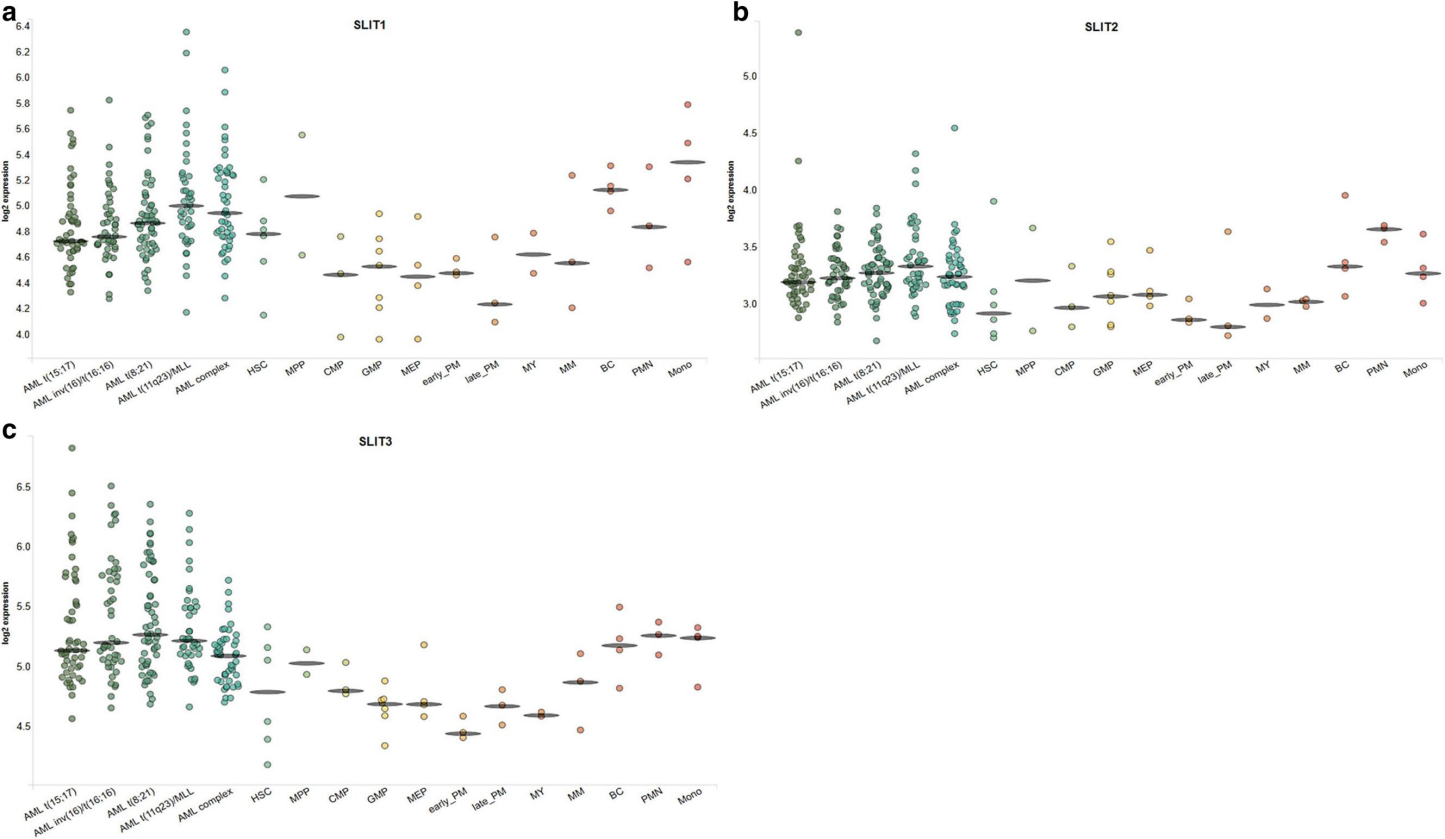
The comparison of mRNA levels between leukemic and normal hematopoietic cells using BloodSpot tool; **a** SLIT1, **b** SLIT2, **c** SLIT3

The in silico analysis using BloodSpot tool revealed differences in higher RNA expression of ROBO3 and ROBO4 in leukemic cells compared to normal bone marrow cells. There was also a tendency for a higher RNA level of ROBO1 and lower expression levels of SLIT1 and SLIT2 among leukemic cells compared to normal bone marrow, whilst there were no clear differences regarding ROBO2 and SLIT3 (Figs. 6, 7).

### Relationship between SLIT–ROBO Expression and Angiogenesis

**Fig. 8:**
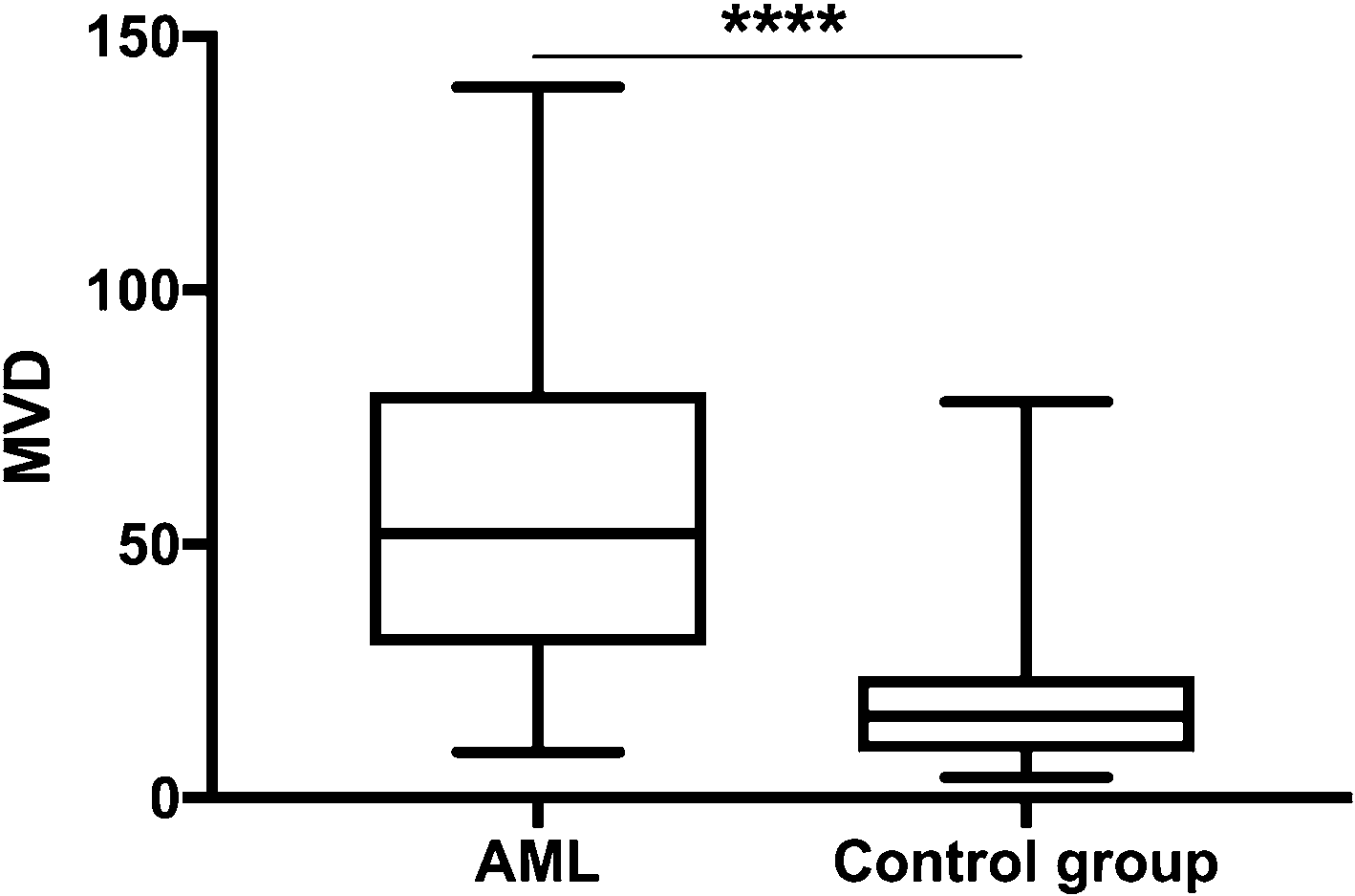
MVD level in AML and the control group

**Fig. 9:**
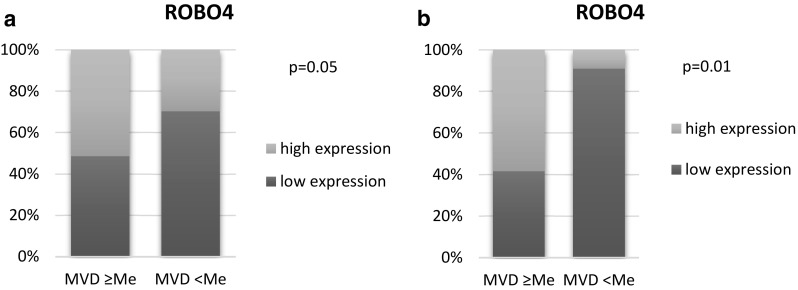
The relationship between ROBO4 expression and MVD level in AML patients (**a**) and in the control group (**b**). The cut-off point for high and low MVD level is the median (Me)

Median MVD in AML samples (51, range 9–140) was significantly higher than in controls (16, range 4–78, *p* < 0.0001). The results are presented in Fig. 8. ROBO4 was the only protein whose expression correlated significantly with MVD. In the AML group, high expression of ROBO4 was more frequently observed in patients with MVD level above the median. The majority of patients with lower MVD had more often low expression of ROBO4 (70.27%). The differences reached statistically significance (*p* = 0.05). The results are presented in Fig. 9a. In addition, in the control group, 58.33% patients with MVD above the median were high expressers of ROBO4, whereas 90.91% of patients with low MVD (below the median) were low expressers of ROBO4 (*p* = 0.01), Fig. 9b. The immunohistochemical staining for CD34 is presented in Fig. 1.

### The Relationship between SLIT–ROBO Expression and Prognostic Factors

We evaluated the relationship between SLIT–ROBO expression and known prognostic factors such as age, cytogenetic risk group, and clinical stage parameters. Higher expression of ROBO1, ROBO2, and ROBO3 was more often observed in AML patients aged ≥ 60 years (*p* = 0.04, *p* = 0.008, and *p* = 0.02, respectively). Conversely, low ROBO4 expression was more often observed in elderly AML (*p* = 0.06). The majority of patients with de novo AML had low SLIT1 (*p* = 0.05) and SLIT2 (*p* = 0.05) expression. In addition, the secondary AML patients were more likely to demonstrate higher ROBO2 expression than control patients although it did not reach statistical significance (*p* = 0.09). High ROBO4 expression was associated with lower WBC in comparison with low expression (2.55 G/l vs 6.31 G/l, *p* = 0.04). No significant correlations were found between other SLIT–ROBO protein expression and either cytogenetic risk group, and clinical stage parameters such as WBC, hemoglobin level, proportion of leukemic blasts in BM, or LDH activity.

**Fig. 10:**
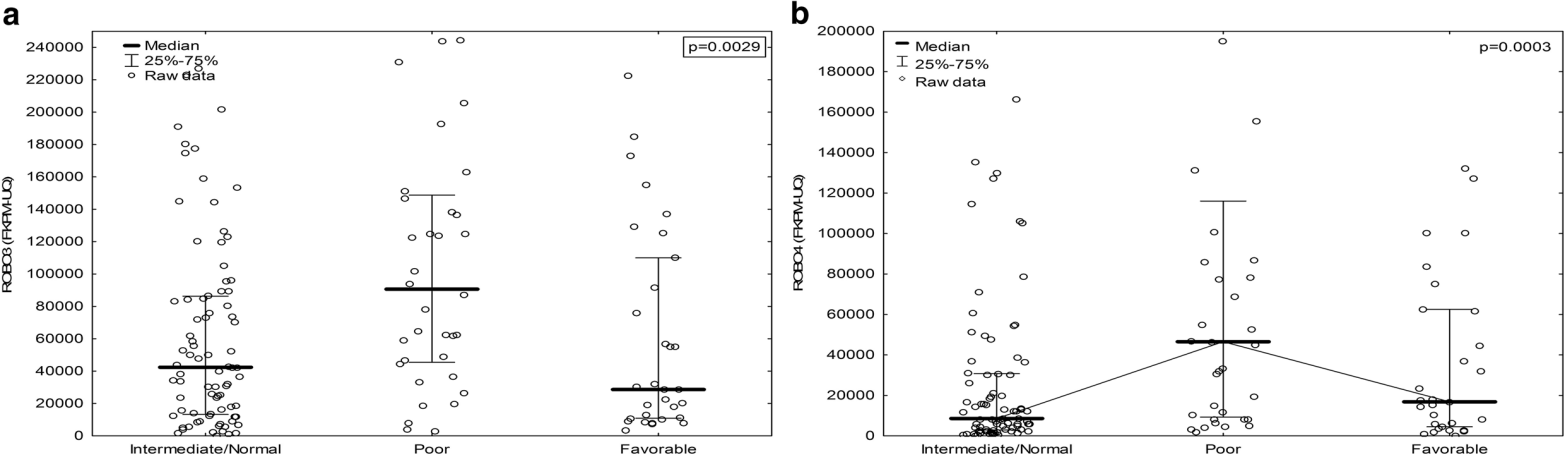
The comparison of ROBO3 and ROBO4 RNA levels regarding CALBG cytogenetic risk groups **a** ROBO3, **b** ROBO4

The in silico analyses for associations between ROBO–SLIT RNA expression levels and clinicopathological data downloaded from The Cancer Genome Atlas Research Network (TCGA) showed some significant results and tendencies. We noted a positive correlation between age of patients and ROBO3 RNA level (*R*2 = 0.30, *p* < 0.05), and we noted negative correlations between ROBO1, ROBO2, SLIT2 and SLIT3 RNA levels (*R*2 = − 0.44, *R*2 = − 0.26, *R*2 = − 0.28, *R*2 = − 0.35, respectively, *p* < 0.05). High expression levels of ROBO3 and ROBO4 RNA were noted in poor Cancer and Leukemia Group B (CALGB) cytogenetic risk group of patients in comparison with normal and favorable risk groups (*p* = 0.0029 and *p* = 0.0003, respectively, from ANOVA-Kruskall–Wallis test) (Fig. 10).

### The Relationship between SLIT–ROBO Expression and Clinical Outcome

No significant difference in CR rate was found between low and high expressers of any of the SLIT–ROBO proteins in the intensively treated group (*n* = 37). Similarly, SLIT–ROBO protein expression influenced overall survival (OS), neither in intensively nor in non-intensively treated AML patients.

**Fig. 11:**
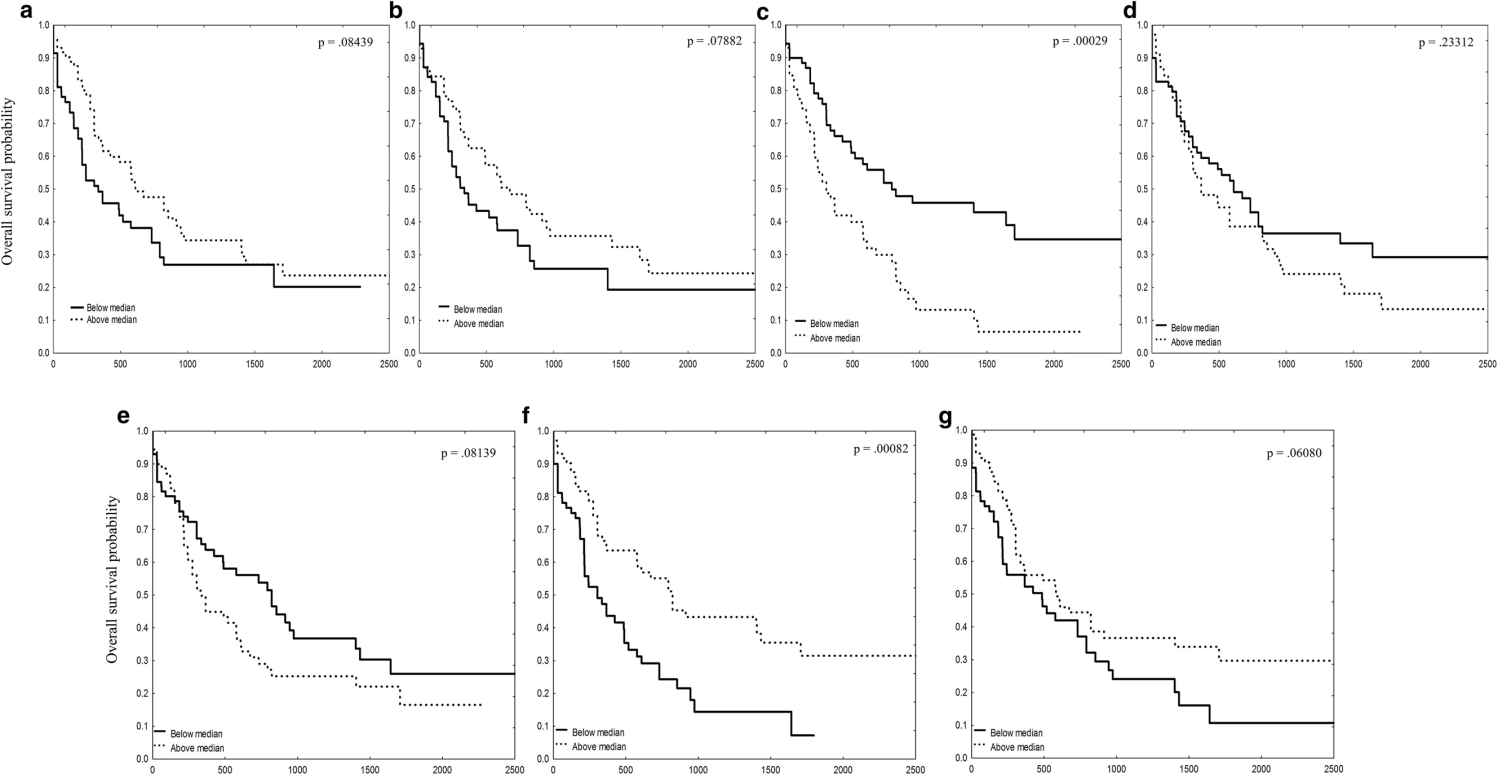
The Kaplan–Meier curves for overall survival (OS) comparison between patients with SLIT–ROBO mRNA expression levels above and below median. Time is given in days. *p* values were calculated using log-rank test; **a** ROBO1, **b** ROBO2, **c** ROBO3, **d** ROBO4, **e** SLIT1, **f** SLIT2, **g** SLIT3

In the in silico analysis of TCGA data, we created Kaplan–Meier plots for OS comparison between patients with RNA levels below vs above median of each gene from SLIT–ROBO RNA. These analyses showed two significant associations. First, patients with high ROBO3 RNA levels had poorer OS in comparison with patients with low ROBO3 RNA levels (*p* = 0.0003). Second, patients with low SLIT2 RNA levels had poorer OS in comparison with patients with high SLIT2 RNA levels (*p* = 0.0008) (Fig. 11).

## Discussion

Among the neuronal cues involved in angiogenesis, the SLIT–ROBO family has been extensively investigated. Numerous studies have indicated its clinical relevance in solid tumors such as endometrial carcinoma, cervical cancer, or breast cancer (Schmid et al. 2007; Ma et al. 2010; Mitra et al. 2012). However, little is known about the neuronal guidance molecules in hematological malignancies. Until now, only few studies have addressed the role of ROBO4 in AML (Chen et al. 2015; Wellbrock et al. 2012). To the best of our knowledge, this study is the first to evaluate the expression of entire SLIT–ROBO protein family in AML.

Expression of ROBO–SLIT proteins in hematopoietic cells is still insufficiently known. We observed the expression of ROBO–SLIT proteins in BM of AML patients and controls in all lines mainly in myeloid cells and megakaryocytes. According to the Human Protein Atlas, ROBO1 and ROBO2 are highly expressed with strong intensity in normal hematopoietic cells in cytoplasmic/membranous location. The expression of ROBO 3 and ROBO4 is not determined in the aforementioned source. The expression of SLIT 2.3 was described as various from low to high with moderate and strong intensity in cytoplasmic/membranous/nuclear location, but the expression of SLIT 1 is not determined in normal bone marrow (http://www.proteinatlas.org^2018^). According to the literature, the expression may also be present in HSC (Smith-Berdan et al. 2011), what could be best revealed by double IHC staining or in the flow cytometry (FCM) method. Our study was based on the IHC analysis of bone marrow sections comparing tumors with healthy controls. The assessment was semi-quantitative evaluating the overexpression of the tested proteins, which could be more objectively assessed on the RNA level. That is why we run the in silico analyses using the Bloodspot tool as described in the Methods. The results confirmed a part of our findings, but still a larger study focusing on RNA level is warranted as the data on the Bloodspot website was restricted to limited number of probes which investigated selected genes in unaffected bone marrow cells. According to the literature, there might be some discrepancies between RNA and protein expression in ROBO–SLIT family. Unlike SLIT3, in which antibody staining is mainly consistent with RNA expression data, antibody staining for ROBO1 and SLIT2 tends to be not consistent with RNA expression (http://www.proteinatlas.org^2018^). However, in Chen et al. study Robo4 protein expression measured by scoring IHC of BM biopsy specimens correlated with Robo4 mRNA expression in the 30 patients studied (Chen et al. 2015). Therefore, it was important to evaluate the expression of SLIT–ROBO proteins in comparison with the cell morphology and their location in the bone marrow in trephine biopsy.

We observed significantly higher expression of ROBO1 and ROBO2 in BM of AML patients compared to the control group, while no significant differences were found in the expression of ROBO3 and ROBO4 between the groups. Interestingly, study of Xu et al. revealed low expression of *ROBO1* and *ROBO2* in leukemia cell lines (Xu et al. 2015). The transfection of the lines with ectopic ROBO1 or ROBO2 caused above 400-fold increased expression of the receptors and resulted in inducing apoptosis of the cells. The effect was absent if mutants ROBOs were transferred. Additionally, using whole-exome and targeted sequencing in 209 patients with myelodysplastic syndrome (MDS) found *ROBO1* and *ROBO2* mutations in 26 (12.4%) patients (Xu et al. 2015). From these, 13 patients had paired samples at both, lower and higher stages of MDS. As a result, 4 out of 13 patients (30.8%) had acquired a *ROBO1* (*n* = 2) or *ROBO2* (*n* = 2) mutation during disease progression. The analysis in acute leukemia cell lines revealed no mutations in either of the genes. It would be interesting to assess the *SLIT* expression level to know if the expression of the ligand or receptor is linked to each other in acute leukemia. A hypothesis that *SLIT* negatively regulates the *ROBO* expression in solid cancers was proposed by Tie et al. (2010). The most frequent described alteration of *SLIT* in tumorigenesis is loss of heterogeneity (LOH) (Singh et al. 2007). As a consequence, micro-RNA(miR) fragments: miR 218-1 and miR 218-2, which are localized in *SLIT2* and *SLIT3* introns, are negatively regulated. In turn, the regulatory role of the miRs on *ROBO1* expression is impaired causing overexpression of the latter. It was proven in cervical, breast, gastric cancers, and small cellular lung cancer (Dickinson et al. 2004, 2010; Tie et al. 2010). In our study, low expression of SLIT1, SLIT2, and SLIT3 ligands was found more often in the AML than in the control samples. Our results may support the hypothesis. In another study, *ROBO1, ROBO4*, and *SLIT2* mRNA expression were quantitatively assessed using RQ-PCR in 104 AML patients (Wellbrock et al. 2012). The authors decided not to determine cut-off level for expression of *ROBO1* and *SLIT2*, instead of that the cohorts were divided according to qualitative feature (positive or negative expression). *ROBO1* expression was observed in 27% of patients. Additionally, the expression of *SLIT2* was revealed also in minority of patients (18%) (Wellbrock et al. 2012). These observations are in accordance with results obtained by Dunwell et al. who examined the methylation status of *SLIT2* in childhood acute lymphoblastic leukemia (ALL) and chronic lymphocytic leukemia (CLL) (Dunwell et al. 2009). *SLIT2* was found to be methylated in 64% of BM samples of ALL and 80% blood samples of CLL. The expression of the gene was restored after treating ALL cell lines with a hypomethylating agent. The samples examined in our study demonstrated low expression of SLIT2 in 74.51% of BM samples of AML but only in 16.67% of BM samples of the control group. In a study of Xu et al. in which ROBO1 and ROBO2 overexpression was arranged by transfecting leukemia cell lines, the addition of exogenous recombinant hSLIT2 led to enhanced apoptosis and inhibited cell growth (Xu et al. 2015). Taken together, these data suggest that the silencing of *SLIT* may be involved in leukemogenesis as a tumor suppressor gene.

In contrast to ROBO1 and ROBO2, we did not find any differences between expression levels of ROBO4 in AML patients and in the control group. High expression of ROBO4 was found in 40.51% of the AML samples vs 59.49% in the control group. Wellbrock et al., using RQ-PCR, revealed *ROBO4* expression in 83% primary AML samples. In addition, in 42 samples, the expression of ROBO4 on the AML blasts was evaluated by fluorescence-activated cell sorting (FACS). The expression of ROBO4 was found in the majority of samples (81%), but it was restricted only to a small proportion of AML blasts. No frequencies of high and low expression were demonstrated. Double staining for CD34 and ROBO4 in FCM showed co-expression of these from 0 to 22% of cells. Authors have claimed that ROBO4 expression is restricted to CD34-sub-population of AML cells (Wellbrock et al. 2012). Chen et al. studied the expression of *ROBO4* mRNA assessed by RQ-PCR in BM samples of 218 patients with AML. Additionally, in 30 BM samples, the expression was further evaluated using immunohistochemistry (IHC) (Chen et al. 2015). Twenty normal BM samples were used as controls. The results revealed higher expression of *ROBO4* mRNA in AML patients than in the control group. The cut-off level of *ROBO4* expression was 0.010. In AML group, the percentage of higher expression revealed 45.4%, versus 54.6% of lower expression. In addition, the authors observed positive correlation of mRNA expression and protein expression in IHC staining. In contrast to the results of Wellbrock et al., higher ROBO4 expression correlated positively with the expression of CD34 on the AML cells (*p* = 0.0009). The percentages of low and high expression level are similar to these in our group. However, one of the limitations of our research is lack of differentiation of AML cell sub-populations which is due to chosen methodology. Interestingly, the correlation with CD34 in both cited articles is disparate. Considering all above results, maybe the expression of ROBO4 is present in majority of AML cells but its level depends on cell sub-population.

The second part of our study evaluated the relationship between SLIT–ROBO family expression and angiogenesis activity. ROBO4 is the first one in the SLIT/ROBO family which was recognized as being involved in angiogenesis (Huminiecki 2000). There is mounting evidence that ROBO4 is expressed at sites of active angiogenesis, both, physiological and pathological (Huminiecki et al. 2002; Wang et al. 2003; Dai et al. 2011). Studies on AML showed higher angiogenesis level measured as MVD in BM with AML than in healthy BM (Padro et al. 2000; Albitar et al. 2001). In accordance with previous studies, our results revealed higher MVD level in the AML group than in the control group (*p* < 0.0001). ROBO4 was the only protein which expression correlated significantly with MVD. Both for AML and control BM samples with higher MVD score demonstrated higher expression of ROBO4 (*p* = 0.05 and *p* = 0.01, respectively). Nevertheless, some research prove anti-angiogenic effect of ROBO4 (Jones et al. 2008; Koch et al. 2011). Due to many contradictory results, the role of ROBO4 and the whole family SLIT–ROBO in angiogenesis requires further investigation. To the best of our knowledge, this is the first study to reveal the correlation between ROBO4 expression and angiogenic activity in BM in AML.

As the karyotype of the AML blasts has a strong influence on the prognosis of AML, the stratification of newly diagnosed AML is based on cytogenetic risk. In our study, SLIT–ROBO protein expression did not differ within the cytogenetic-risk groups. Chen et al. report that low ROBO4 expression was more often observed in AML patients from the intermediate cytogenetic risk (*p* = 0.049) (Chen et al. 2015). However, in the same population, high ROBO4 expression correlated strongly with *t*(8;21) AML, which is considered as favorable risk AML, and low expression of the protein with *t*(15;17) AML. The discordant results may be due to the different regulation of ROBO4 expression in different cytogenetic risk groups. To have a complete view of whether the proteins influence the prognosis, additional studies on larger groups should be performed.

We did not observe any significant correlation between any SLIT–ROBO protein and either the clinical course of the disease, such as CR rate, OS or DFS. This may be due to heterogeneity of our group in terms of treatment regimens. Only 48% received intensive induction treatment, 38% of patients received HMA (hypomethylating agents) or LDAC (low dose cytarabine) and 14% best supportive care only. In the study of Chen et al. high ROBO4 expression was associated with shorter OS (*p* = 0.023) and disease-free survival (DFS) (*p* = 0.024) (Chen et al. 2015). Additionally, patients with high ROBO4 expression tended to achieve lower CR rate than those with low ROBO4 expression. The results confirmed previously presented observations by Wellbrock et al. who revealed that high ROBO4 expression negatively impacts OS (*p* = 0.002) and event-free survival (EFS, *p* = 0.046) (Wellbrock et al. 2012). Similarly, high ROBO4 expression coincided with a lower CR rate. In contrast to our study, only patients below 60 were included to the OS and CR analysis. The in silico analysis using TCGA data repository showed a significant poor risk value of high ROBO3 and low SLIT2 RNA expression, but a larger study is warranted to show the actual prognostic value of SLIT–ROBO family among patients with AML.

In conclusion, our results indicate that although the precise function of SLIT–ROBO family members remains unknown, they might play a role in the biology of AML. Low expression of SLIT1, SLIT2, and SLIT3 and high expression of ROBO1 and ROBO2 in the BM of AML patients may suggest deregulation of SLIT–ROBO pathway in AML. The study is a good starting point for more detailed research to evaluate the results. The further correlation with molecular biology methods may answer on which level of the protein expression the aberration arises and whether it has a prognostic impact. A better understanding of the SLIT–ROBO signaling pathway in leukemic blasts may increase the knowledge about leukemogenesis.
